# Hybrid Macrocyclic Polymers: Self-Assembly Containing Cucurbit[m]uril-pillar[n]arene

**DOI:** 10.3390/polym14091777

**Published:** 2022-04-27

**Authors:** Zhaona Liu, Zhizheng Li, Bing Li, Le Zhou, Huacheng Zhang, Jie Han

**Affiliations:** 1 Medical School, Xi’an Peihua University, Xi’an 710125, China; zhaonaliu@peihua.edu.cn; 2 School of Chemical Engineering and Technology, Xi’an Jiaotong University, Xi’an 710049, China; lizhizheng@stu.xjtu.edu.cn (Z.L.); 3121116050@stu.xjtu.edu.cn (B.L.); zhou1999@stu.xjtu.edu.cn (L.Z.); 3 Key Laboratory of Advanced Energy Materials Chemistry (Ministry of Education), College of Chemistry, Nankai University, Tianjin 300071, China

**Keywords:** self-assembly, hybrid macrocycles, cucurbit[m]uril, pillar[n]arene, host–guest inclusion

## Abstract

Supramolecular self-assembly by hybrid macrocycles containing both cucurbit[m]uril (CB[m]) and pillar[n]arene was discussed and summarized in this review. Due to different solubility, diverse-sized cavities, and various driving forces in recognizing guests, the role of CB[m] and pillar[n]arene in such hybrid macrocyclic systems could switch between competitor in capturing specialized guests, and cooperator for building advanced hybridized macrocycles, by controlling their characteristics in host–guest inclusions. Furthermore, both CB[m] and pillar[n]arene were employed for fabricating advanced supramolecular self-assemblies such as mechanically interlocked molecules and supramolecular polymers. In those self-assemblies, CB[m] and pillar[n]arene played significant roles in, e.g., microreactor for catalyzing particular reactions to bridge different small pieces together, molecular “joint” to connect different monomers into larger assemblies, and “stabilizer” in accommodating the guest molecules to adopt a favorite structure geometry ready for assembling.

## 1. Introduction

With an origin derived from the mimic of natural and biological process [1,2,3,4], supramolecular self-assembly [5,6,7], especially supramolecular polymers [8,9], has provided an interesting research focus [10,11] in recent decades for designing and fabricating marvelous smart materials [12,13,14] via noncovalent interactions [15,16]. Self-assembly, as an important supplementary method to classic organic synthesis and polymerization, has aroused much research interest [17,18]. Particularly, choosing the significant building blocks during preparation has not only affected the functions of supramolecular polymeric self-assembly [19,20], but also has decided the procedure/progress of building such supramolecular architectures [21,22,23]. As one type of important building blocks [22], macrocycles [24,25] and their host–guest inclusions [26] have frequently participated in the procedure of building complicated hierarchical supramolecular polymeric self-assembled materials [27]. Interestingly, to achieve the particular target in function and application [28,29,30], several different kinds of macrocycles [24] have been simultaneously employed in fabricating supramolecular polymeric self-assemblies, leading to the formation of the significant hybrid macrocyclic system [31,32,33].

Actually, the fabrication of a proper hybrid macrocyclic system by diverse macrocycles was not easy [32,34,35]. There were several difficulties in the design strategy and experimental procedure [36]. For example, the modification and functionalization of various macrocycles were usually challenging [37], in addition to coupling them together. If not adopting the proper design and efficient synthesis methods, the building of hybridized macrocycles might become not only overdesigned, but also time-consuming [38,39]. In addition, during the construction, different behaviors of diverse macrocycles in molecular recognition should be controlled and balanced in order to achieve the purpose of function and application [40,41]. For example, the different-sized host cavities [26] and various driving forces [42,43] capturing guests should be paid attention during molecular recognition. Furthermore, the role of diverse macrocycles in a hybrid system should be fully considered and taken advantage of. For example, some macrocycles could promote the solubility of the integrated system [44,45,46,47], while other macrocycles could contribute to catalyzing particular reactions during the process of self-assembly [48,49,50].

**Chart 1:**
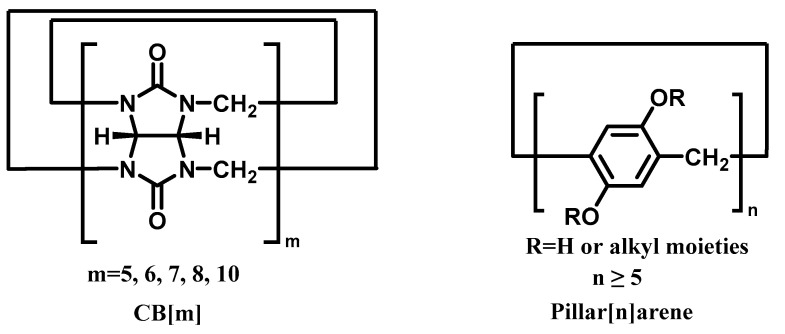
Illustration of chemical structures of CB[m] and pillar[n]arene.

Pillar[n]arene [51,52] (Chart 1) was discovered in 2008 and has been widely used in the process of supramolecular self-assembly [53,54]. Due to its unique physiochemical properties and symmetric structures, pillar[n]arene usually has poor solubility in aqueous solutions [55]. Thus, such a macrocycle requires particular modification to possess the ability of solubilizing in aqueous solutions [56,57,58]. Several functional groups have been involved including carboxylate and ammonium salts [59,60,61,62]. However, the method and choice of dissolving pillar[n]arene in aqueous solutions were limited. It will be interesting to investigate whether other water-soluble macrocycles could integrate with pillar[n]arene in a hybrid system and promote its solubility in aqueous solution or not. One favorite candidate is cucurbit[m]uril (CB[m], Chart 1) [63,64], composing cyclic repeating glycoluril. Furthermore, it will be fantastic to learn whether the addition of CB[m] will play other roles in the hybrid system, or not. It is already known that the hydrophobic cavity of CB[m] can show similar molecular recognition as that of pillar[n]arene towards various guests, and CB[m] is commercially available with different-sized cavities, which can enrich the host–guest interaction in the possible integrated hybrid macrocyclic system [65,66].

In this review, we will discuss and summarize the recent progress in building self-assembly containing both CB[m] and pillar[n]arene. Due to the possession of similar cavities, both present in the integrated hybrid system could show competition in including similar/same guests. Thus, a valuable synthesis/preparation strategy was employed in the hybridized system to balance and control their different molecular recognition. Except for competition, the relationship between them in an integrated system also shows cooperation in accommodating diverse guests, in addition to providing the possibility of bridging smaller pieces together for hierarchical self-assemblies such as mechanically interlocked molecules [67,68,69,70,71] and supramolecular polymers [72,73,74,75]. Interestingly, due to the particular structure and physiochemical properties, CB[m] could play diverse roles in the CB[m]-pillar[n]arene hybrid macrocyclic system such as acting as “microreactor” and molecular “joint” [76,77,78]. Finally, because the self-assembly containing CB[m]-pillar[n]arene hybrid macrocyclic system is still under development, we will try to raise some scientific and technical issues in this review, and propose considerable challenges for future research.

## 2. The Competition and Cooperation between CB[m] and Pillar[n]arene as the Host

As significant hosts, both CB[m] and pillar[n]arene could show different characteristics in host–guest complexation, due to diverse-sized cavities and various driving forces in recognizing guests. Interestingly, both could exhibit very strong capacity in molecular recognition towards the same guest. Thus, if placed under specialized conditions, they could definitely show competition in efficiently capturing those particular guests. Further effort has been made in controlling and balancing their diverse performances in molecular recognition by building integrated self-assemblies, i.e., hybrid macrocyclic systems.

**Scheme 1:**
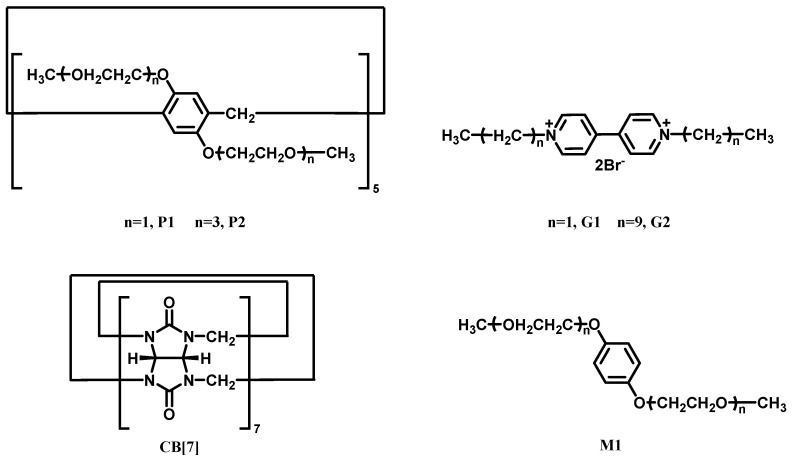
Chemical structures of pillar[5]arene **P1** and **P2**, **CB[7]**, guest molecules **G1** and **G2**, and monomer **M1**.

**Figure 1:**
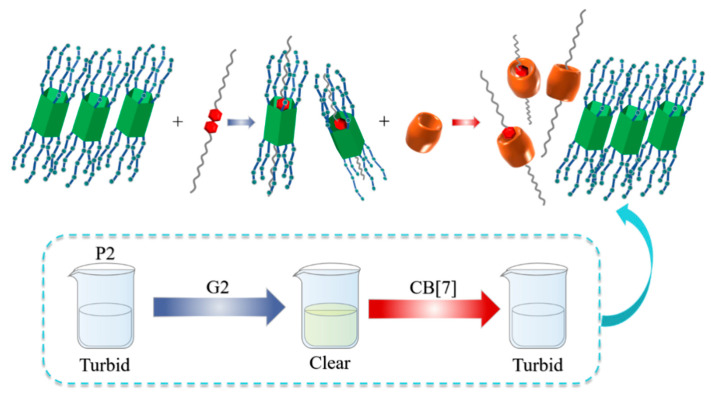
The host–guest inclusion controlled reversible turbid-to-clear/clear-to-turbid transition could be obtained by subsequently adding **G2** (b, 1 equiv.) and **CB[7]** (c, 2.5 equiv.) into the aqueous solution containing **P2** (a, 1 equiv.) at 55 °C. Adapted from [79].

Due to the lock-and-key principle in supramolecular chemistry [79,80], CB[m] and pillar[n]arene usually exhibit different behaviors in recognizing guest molecules with diverse associate constants, (*Ka*). Thus, CB[m] could be used as an extra tool to control the physiochemical properties of self-assembled pillar[n]arene and its host–guest complexes. For example, Ref. [79], in comparison with the monomer **M1** (Scheme 1) and short ethylene oxide chains-bearing pillar[5]arene (**P1**, Scheme 1), pillar[5]arene perfunctionalized with ten outer triethylene oxide groups (**P2**, Scheme 1) could not only have improved solubility in water, but also exhibited the lower critical solution temperature (LCST) behavior [81], i.e., sharp transition and narrow hysteresis (2 °C), due to the possession of both longer oligomeric ethylene oxides and macrocyclic skeletons [79]. Additionally, instead of shorter viologen salts such as **G1** (Scheme 1), **P2** could include the didecylviologen salt (**G2**, Scheme 1) in the stoichiometry of 1/1 with the *Ka* as (4.3 ± 0.5) × 10^3^ M^−1^ at 25 °C, due to stronger hydrophobic and charge transfer interactions. Interestingly, the clouding point (T*_cloud_*) of **P2** could be controlled by the concentration and amount of **G2**, changing from 25 to 60 °C. Particularly, due to that cucurbit[7]uril (**CB[7]**, Scheme 1) could include viologen salts also in 1/1 molar ratio but with the stronger *Ka* as high as 105 × M−1, CB[7] was used a competitive host to exclude **G2** from **P2** (Table 1), leading to the decrease of T*_cloud_* in the system containing **G2** and **P2** to 47 °C [79]. Furthermore, the host–guest inclusion controlled reversible turbid-to-clear/clear-to-turbid transition could be achieved by the subsequent addition of **G2** and **CB[7]** in the solution of **P2** (Figure 1). Actually, both CB[m] and pillar[n]arene could fully utilize their capacities of capturing guests to further affect the morphologies of guests-based self-assemblies [82].

**Scheme 2:**
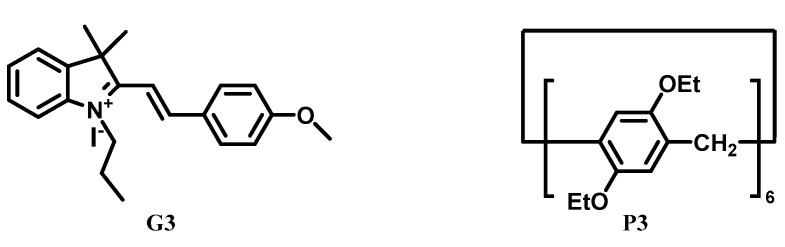
Chemical structures of pillar[6]arene **P3** and **G3**.

Because of the different-sized cavities, CB[m] and pillar[n]arene could also adjust their own cavities with proper sizes, i.e., choose the pair of hosts with proper sized cavities, to present the similar *Ka* towards the specialized guest. However, they might have different types of host–guest inclusions in accordance with diverse driving forces. For example, with the similar *Ka* of around 10^4^ × M^−1^, **CB[7]** (Scheme 1) could form inclusion with the guest of hemicyanine dyes (**G3**, Scheme 2) in the stoichiometry of 1/1 due to the ion–polar interaction, while pillar[6]arene **P3** (Scheme 2) could interact with **G3** in 2/1 molar ratio with a sandwiched binding model due to the intermolecular interaction with the electron donor–acceptor, i.e., exo-wall complexation, where the guest was not included into the cavity of **P3 [83]**.

**Scheme 3:**
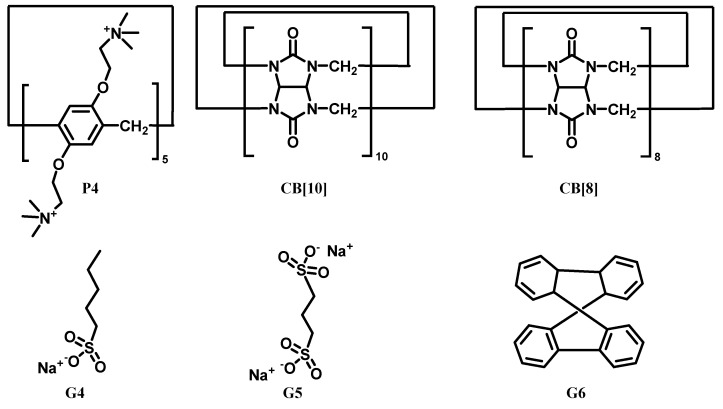
Chemical structures of pillar[5]arene **P4**, **CB[10]** and **CB[8]**, and **G4-G6**.

**Figure 2:**
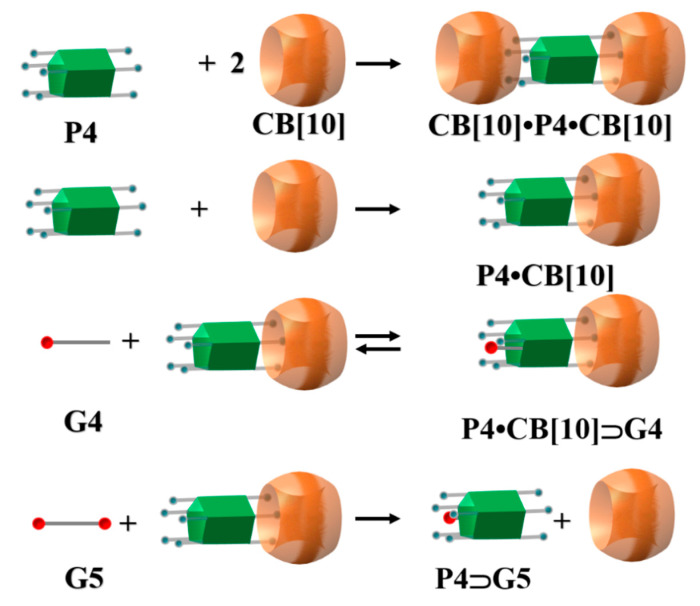
Hybrid macrocyclic systems **P4•CB[10]** were prepared in diverse molar ratios of 1/2 and 1/1, and the different performances of 1/1 **P4•CB[10]** in recognizing **G4** and **G5**.

Thus, by taking advantage of their own characteristics such as various *Ka* and binding model in recognizing guests, CB[m] and pillar[n]arene could cooperate together to interact with guest molecules in an integrated hybrid macrocyclic system. For example, ref [84], due to the ion-dipole and CH^…^O interactions as indicated by computational studies [85] using a semi-empirical tight binding method, the ring-on-ring hybrid macrocyclic host systems were just prepared by integrating **CB[10]** and polycationic perfunctionalized pillar[5]arene **P4** (Scheme 3) together, where the complex ratio could be tuned from 1/2 to 1/1 (Figure 2), as confirmed by ^1^H NMR titration. Interestingly, the weaker interaction between **P4** and **CB[8]** (Scheme 3) further revealed that the size match between pillar[n]arene and CB[m] was also vital for successfully building a hybrid macrocyclic composite. Particularly, characteristics in recognizing various guests by both cavities of **CB[10]** and pillar[5]arene could be further controlled and balanced, leading to either the formation of complicated multiple complexes or the damage of the hybrid macrocyclic host systems (Table 1). Due to the possession of two independent hybrid cavities (Figure 2), both 1-pentanesulfonate (**G4**, Scheme 3) and 9,9′-spirobifluorene (**G6**, Scheme 3) could be included by the hybrid macrocycles via the cavities of pillar[5]arene and **CB[10]**, exhibiting strong *Ka* as (3.8 ± 0.4) × 10^6^ and (3.3 ± 0.7) × 10^5^ M^−1^, respectively. However, the butane-1,4-disulfonate (**G5**, Scheme 3) could trigger the disassembly of hybrid macrocycles P4•**CB[10]**, and precipitate **CB[10]** in the solution phase (Figure 2).

## 3. The Preparation of Mechanically Interlocked Molecules Containing CB[m] and Pillar[n]arene

Except for utilizing their diverse characteristics for promoting competition and cooperation in molecular recognitions, both CB[m] and pillar[n]arene were further employed for fabricating advanced mechanically interlocked molecules (MIM) by fully considering the CB[m]-catalyzed alkyne-azide 1,3-dipolar cycloaddition, namely, “click” reaction [86,87], in addition to the strong capacity of pillar[n]arene in accommodating and stabilizing specialized linear cationic substrates [88]. In other words, the molecular “rod” in building MIM could be captured by enough pillar[n]arene moieties with an expected rigid and linear geometry, while CB[m] could hold the molecular “stoppers” and promote the formation of the “bridge” between the “rod” and “stoppers” by “click” reactions. Finally, both CB[m] and pillar[n]arene acted as the “wheel” in the obtained MIM.

**Scheme 4:**
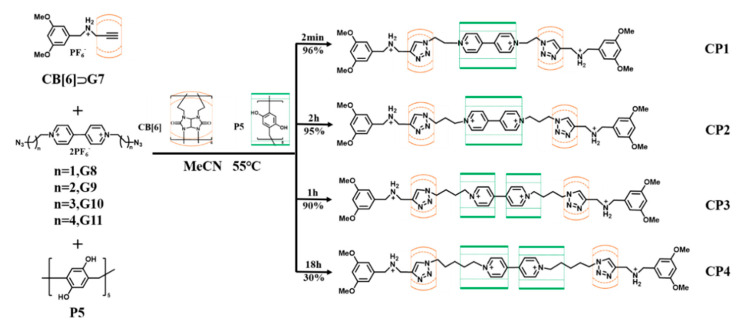
The synthesis of dumbbell compounds **CP1-CP4** by using **G7-G11**, **P5** and **CB[6]**.

**Scheme 5:**
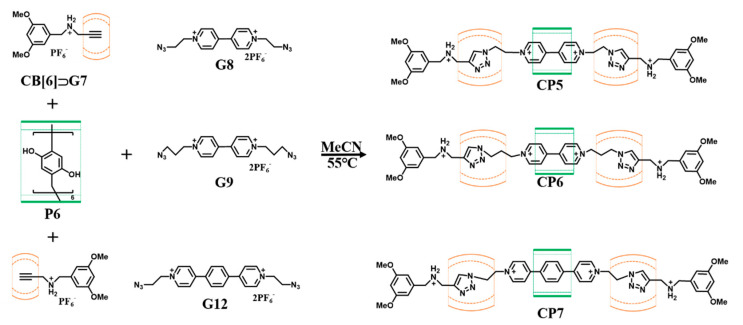
The synthesis of dumbbell compounds **CP5-CP7** by using **G8**, **G9** and **G12**, **P6** and **CB[6]**.

**Figure 3:**
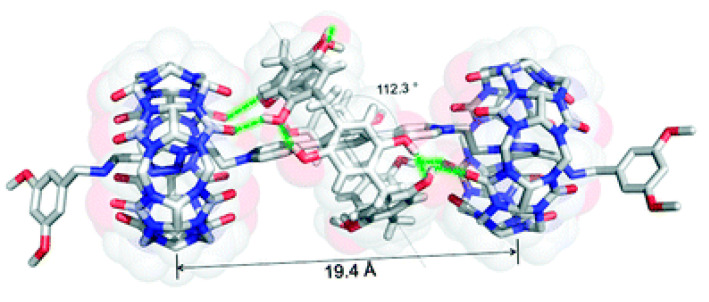
X-ray single crystalline structures of **CP7** in solid state. The important intermolecular hydrogen bonds are highlighted by using green dash lines. All hydrogen atoms not used in the formation of hydrogen bonding were omitted for clarity [90]. (Adapted and reprinted with permission from [90] © 2014, *Chemical Communications*).

For example, Ref. [89], two [4]rotaxanes such as **CP1** and **CP2** and two [5]rotaxane **CP3** and **CP4** (Table 2) were cooperatively prepared in the presence of the host–guest inclusion of **CB[6]**⸧N-(3,5-dimethoxybenzyl)propargylammonium chloride (**G7**, Scheme 4), pillar[5]arene (**P5**, Scheme 4) and linear azidoalkyl-modified bipyridinium dications such as **G8-G11** (Scheme 4) via **CB[6]**-catalyzed “click” reaction at 55 °C in acetonitrile with the yield ranging from 30–96%. With similar design strategy and synthesis methods by using the stopper with CB[m] as the catalyst—**CB[6]⸧G7**, in addition to the “rod” of **G8**, **G9** and **G12 [88]**, pillar[5]arene moiety was further substituted by pillar[6]arene (P6, Scheme 5), leading to the synthesis of different dumbbell compounds including **CP5-CP7** (Scheme 5 and Table 2) in the yield of 39–68% [90]. Interestingly, due to the lock of pillar[5]arene and pillar[6]arene in those space limited dumbbell compounds, in addition to the particular functional groups such as carbonyl and hydroxyl moieties rooted in CB[m] and pillar[n]arene, respectively, [4]rotaxane possessed various interesting conformational isomers with the assistance of forming intermolecular hydrogen bonds (Figure 3) [88,89,90,91,92].

## 4. The Preparation of Supramolecular Polymer containing CB[m] and pillar[n]arene

As shown in the above examples, the CB[m]-catalyzed “click” reactions were able to produce the “angular” chemical structures, acting as the “stopper” in fabricating the space-limited self-assemblies such as MIM. If preparing infinite polymeric structures, both CB[m] and pillar[n]arene mainly performed as the molecular “joint” to accommodate linear guests and direct the structural geometry of larger supramolecular assemblies.

**Scheme 6:**
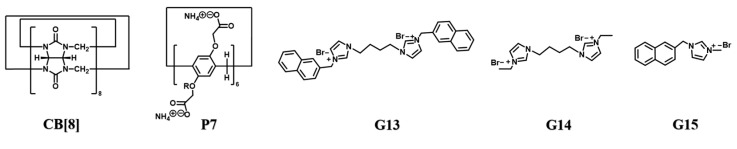
Chemical structures of pillar[6]arene **P7**, and guests including **G13–G15**.

**Figure 4:**
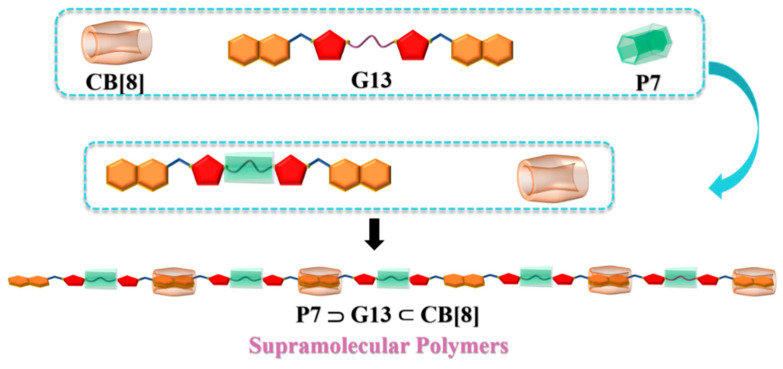
The formation of supramolecular polymers **P7⸧G13⊂CB[8].** Adapted from **[93]**.

For example, water soluble carboxylate perfunctionalized pillar[6]arene (**P7**, Scheme 6) exhibited selected molecular recognition towards imidazolium salt **G14** and **G15** bearing different groups with different *Ka* of 1.31 × 10^6^ and 6.39 × 10^5^ M^−1^, respectively. Thus, **P7** could further complex with imidazolium subunits on a specialized guest with naphthalene moieties on both ends (**G13**, Scheme 6), leaving the naphthalene subunits to be captured by **CB[8]**. Due to the enhancement of π-π stacking interactions of two nearby naphthalene groups by **CB[8]**, supramolecular polymers **P7⸧G13⊂CB[8]** (Figure 4 and Table 2) could be prepared by those ternary components [93].

## 5. Overview and Outlook

In conclusion, supramolecular self-assembly based on hybrid macrocyclic systems containing pillar[n]arene and CB[m] were summarized in this review. Both CB[m] and pillar[n]arene are significant hosts in the formation of host–guest complexation. Their behaviors in molecular recognition could be greatly affected by several issues such as solubility in various solutions, various cavities and different driving forces in complexing guests. Both could exhibit strong competition in complexing the same/similar favorite guest molecules. Except for competition, the capacity of CB[m] and pillar[n]arene in host–guest inclusion could be integrated into the hybrid macrocyclic system in order to enhance their performance in recognizing specialized guests. Furthermore, the structural skeletons of CB[m] and pillar[n]arene were employed for fabricating advanced supramolecular self-assemblies such as MIM and supramolecular polymers. In those self-assembled systems, hybrid hosts served as: 1) the molecular “joint” to connect different small molecular moieties together into larger assemblies; 2) the “microreactor” to catalyze specialized reactions which could further covalently couple building blocks into rigid architectures; and, 3) the “stabilizer” to accommodate and stabilize guest molecules to adopt favorite molecular geometry for advanced and complicated self-assembly.

Although much important work has been conducted in this area, several issues should be paid attention in future studies, for example:

(1) More hybrid macrocyclic systems should be prepared in order to accomplish advanced chemical structures in addition to applicable functions, such as with new design strategy and new components. To date, pillar[n]arene derivatives have rarely been used as a type of effective supplement [94] for controlling CB[m]-based supramolecular systems by taking advantage of the particular external stimuli responsiveness of pillar[n]arene. In addition, several types of CB[m] have been used in preparation procedures, but only pillar[5/6]arene has been employed in the construction of a CB[m]-pillar[n]arene hybrid macrocyclic system. Actually, the performance of pillar[n]arene with larger cavities has more interesting behavior in host–guest complexes [95,96,97]. If they could be involved in the fabrication, the hybrid system will definitely possess different functions and “selling” points.

(2) Advanced and more complicated self-assemblies such as micelles and vesicles could be further prepared by taking advantage of different models such as self-assembled amphiphiles [98], which could be of interest in exploring the functions of cargo-delivering [99,100,101]. Furthermore, except for rotaxane, catenane [102,103,104,105] could also be prepared by integrating both pillar[n]arene and CB[m], enriching the family of MIM with mechanical bonds.

(3) Applications of CB[m] and pillar[n]arene-containing hybrid macrocyclic systems should be explored further. Currently, after forming inclusions in building self-assemblies such as MIM and supramolecular polymers, the cavities of pillar[n]arene and CB[m] will be fully occupied and have no further function. If those cavities in self-assembled systems could be freed, they might, accordingly, accomplish more interesting applications, such as for biomedicines, in addition to adsorption and separation in specialized industry [106,107,108].

## Figures and Tables

**Table 1:** The relationship between CB[m] (**CB[7]** and **CB[10]**) and pillar[n]arene (**P2** and **P4**) including competition in acting as the host towards guests such as **G2**, and cooperation in building hybrid macrocyclic system to include guests such as **G4-G6**.

CB[m]	Pillar[n]arene	Relationship	Guests	Ref
**CB[7]**	Pillar[5]arene **P2**	Competition of hosts (*Ka* of **P2**⸧**G2** ~10^3^ M^−1^) vs. (*Ka* of **CB[7]**⸧**G2** ~10^5^ M^−1^)	**G2**	[79]
**CB[10]**	Pillar[5]arene **P4**	Forming hybrid macrocyclic system **CB[10]•P4** presenting integrated *Ka* towards **G4** and **G6** as ~10^6^ and ~10^5^ M^−1^	**G4**-**G6**	[84]

**Table 2:** Self-assembled hybrid macrocyclic systems such as [n]rotaxane **CP1-CP7** and supramolecular polymer **P7⸧G13⊂CB[8]** by different CB[m] such as **CB[6]** and **CB[8]**, pillar[n]arene including **P5-P7**, and guests including **G7-G13,** as the important building blocks.

CB[m]	Pillar[n]arene	Self-Assembly	Guests as Building Blocks	Ref
**CB[6]**	Pillar[5]arene **P5**	n]Rotaxane **CP1-CP4**	**G7-G11**	[89]
**CB[6]**	Pillar[6]arene **P6**	[n]Rotaxane **CP5-CP7**	**G8**, **G9** and **G12**	[90]
**CB[8]**	Pillar[6]arene **P7**	Supramolecular polymer **P7⸧G13⊂CB[8]**	**G13**	[93]

## Data Availability

Not applicable.

## Abbreviations

CB[m]	Cucurbit[m]uril
*Ka*	Associate constants
LCST	Lower critical solution temperature
MIM	Mechanically interlocked molecules
T*_cloud_*	Clouding point
